# The effects of financial spatial structure on household financial vulnerability: Evidence from China

**DOI:** 10.1371/journal.pone.0313189

**Published:** 2024-11-01

**Authors:** Hang Gao

**Affiliations:** 1 School of Economics, Capital University of Economics and Business, Beijing, China; 2 Beijing Municipal Institute of Economic and Social Development, Beijing, China; University of Almeria: Universidad de Almeria, SPAIN

## Abstract

Based on the panel data of Chinese Family Panel Studies (CFPS) and cities from 2012 to 2020, this paper explores the impact of financial spatial structure on household financial vulnerability and the moderating effect of financial regulation and financial technology from the perspective of the “local market effects” and “spatial spillover effects” of finance. It is indicated that: firstly, the “local market effects” and “spatial spillover effects” of financial spatial structure effectively alleviate household financial vulnerability and the conclusion is still hold true after conducting endogeneity analysis and a series of robustness test. Secondly, promoting household entrepreneurship, optimizing asset allocation, and enhancing residents’ financial literacy are crucial channels through which financial spatial structure influences household financial vulnerability. Thirdly, financial regulation helps to build a fair and transparent financial market, thereby strengthening the positive effects of “local market effects” and “spatial spillover effects” of finance on household financial vulnerability. Financial technology has improved the quality and efficiency of traditional financial services, helping to further leverage the “local market effects” of finance, while it has no obvious impact on strengthening the “spatial spillover effects” of finance. By subdividing the application areas of financial technology, it is observed that the digitalization of payment and insurance businesses can help alleviate household financial vulnerability through the “spatial spillover effects” of finance.

## 1. Introduction

In recent years, the leverage ratio of the Chinese household sector has been steadily increasing, resulting in a continuous exacerbation of household financial vulnerability. Specifically, from 2015 to 2022, the average leverage ratio of the Chinese household sector (measured as the ratio of household debt to GDP) was 61.9%, accounting for over twenty percent of the macro leverage ratio (data sourced from the Research Center for National Balance Sheet). While appropriate liabilities can facilitate smooth consumption, elevate the household happiness index and enhance overall family well-being [1], over-indebtedness can not only amplify household financial risks but also affect financial market stability, potentially evolving into systemic financial risks [2,3]. Consequently, the question of how to reduce household financial vulnerability and further achieve a dynamic balance between maintaining steady growth and mitigating risks has attracted widespread attention from various sectors of society.

Finance, as the fundamental institution in the development of modern economic society, has increasingly permeated people’s daily lives and significantly influenced household financial vulnerability. The development of financial markets, on the one hand, affects the accessibility of household credit, thereby impacting their financial pressures and vulnerability. On the other hand, it provides a plethora of investment options and opportunities, reducing the costs associated with household entrepreneurship, enhancing the likelihood of entrepreneurial success for residents, and ultimately boosting household income [4]. Since 2015, in response to the evolving stage of economic development, the Chinese government has introduced the concept of supply-side structural reform. In the subsequent year, 2016, the Political Bureau of the CPC Central Committee formally announced its intention to mitigate economic and financial risks, with a particular emphasis on curbing asset bubbles. This announcement signaled China’s official entry into a financial deleveraging cycle, characterized by a combination of "monetary policy tightening" and "strict supervision". Further advancing this reform trajectory, in 2019, the Chinese government outlined a strategic initiative aimed at "further deepening supply-side structural reforms within the financial sector". This directive has notably augmented the role of finance in bolstering the real economy, thereby inaugurating a new era of financial contribution and integration with fundamental economic principles. The Sixth Central Financial Work Conference of China, held in 2023, reiterated the commitment to deepening the structural reform of the financial supply side, emphasizing risk prevention and control as a persistent theme in all financial undertakings.

The optimization of financial structure serves as a crucial indicator of the development of financial markets [5]. In reality, due to factors such as increased transaction costs and declined liquidity caused by geographical distances, coupled with the influence of the "scale effect" and "external economies", the financial spatial structure often exhibits an uneven distribution of financial resources within a specific region, a phenomenon known as financial agglomeration. Globally, an international finance center has emerged, with New York and London serving as its axis. Similarly, in China, financial resources are progressively concentrating in cities that receive policy support. According to the China Financial Center Index (CFCI) Report, the cumulative value-added of the financial industry in 36 national and regional financial centers reached 5.69 trillion yuan in 2021, accounting for 62.4% of the country’s total. Aside from the development of regional financial centers around provincial capitals, financial resources in other prefecture-level cities also tend to be highly concentrated within specific urban spatial areas. A financial spatial structure, characterized by regional financial centers as the primary focus and local finance as a supplementary element, is emerging and continually strengthening. The pertinent question arises: Does the financial spatial structure impact household financial vulnerability, and if so, how? Addressing this query not only aids in comprehending the underlying causes of household financial vulnerability but also offers policy insights for mitigating household financial risks and advancing the development of a modern financial system.

While existing literature on the influencing factors of household financial vulnerability provides valuable theoretical references, it predominantly focuses on the micro-level impact of family householder characteristics and environment on financial vulnerability. Limited research explores the macro-level factors influencing household financial vulnerability, and the effect of financial structure on this aspect remains unexplored by academia. As finance increasingly permeates people’s lives, more individuals engage in diverse financial activities such as investing, saving, borrowing and lending, and insurance. Finance has become a stabilizing force for social equity and individual well-being. To enhance the prevention of household financial risks and maintain a dynamic balance between growth and risk mitigation, it is imperative to establish a logical connection between financial structure and household financial vulnerability. As the financial structure evolves, so does the internal and external environment of the financial industry. This includes the gradual improvement of financial regulations, evidenced by the promulgation of various regulations and rules by the Chinese State Council, such as the Regulations on the Supervision and Administration of Non-bank Payment Institutions and the Administrative Measures for the Capital of Commercial Banks. Additionally, the emergence of financial technology and innovative products, like the "Postal E-chain" by China Postal Savings Bank and "Yu’E Bao" and "JD Finance" by internet platform companies, is continually shaping the financial landscape. While promoting high-quality financial development, these transformations undoubtedly impact the economic ramifications of the financial structure. Therefore, when assessing the influence of the financial structure on household financial vulnerability, it is essential to consider these ongoing changes.

As a global economic powerhouse, China’s financial market reforms not only reshape its domestic economic landscape but also exert profound influence on the global economy. The reform on the financial supply side has ushered in substantial changes in the structure of China’s financial market, marked by significant regulatory reforms and the introduction of innovative financial instruments. This provides a unique perspective to examine the impact of financial structure on household finances and the moderating effects of external financial environment changes. Consequently, we utilize panel data from the Chinese Family Panel Studies (CFPS) and data on Chinese cities spanning from 2012 to 2020 as our research sample to delve into the effect, mechanism, and guarantee conditions of financial spatial structure on household financial vulnerability. Regarding financial spatial structure, we focus on the “local market effects”, measured by local financial geographic density, and the “spatial spillover effects”, measured by the construction of regional financial centers. The regression results indicate that “local market effects” and “spatial spillover effects” of finance significantly reduce household financial vulnerability by promoting household entrepreneurship, optimizing household asset allocation, and enhancing residents’ financial literacy. Furthermore, the strengthening of regional financial regulation and the development of financial technology can reinforce the impact of the financial spatial structure on household financial vulnerability.

Compared to existing research, this paper makes marginal contributions in the following three aspects. Firstly, we introduce a fresh perspective on the causes of household financial vulnerability by examining the role of financial geography. Drawing upon the spatial distribution characteristics of financial resources in China, we clarify how the financial spatial structure impacts household financial vulnerability. This fills a significant gap in previous research, which often overlooked the influence of financial geography factors. Secondly, we present novel empirical evidence that examines the economic consequences of financial spatial structure on the household sector. We pinpoint how the "local market effects" and "spatial spillover effects" of finance affect household financial vulnerability. Additionally, we conduct mechanism tests focused on fostering entrepreneurship, optimizing household asset allocation, and bolstering residents’ financial literacy. These findings offer new empirical insights into understanding the economic implications of financial spatial structure. Finally, our study provides valuable guidance for governments seeking to promote high-quality development in the financial sector. In China, implementing finance for the people and harnessing high-quality financial services to enhance people’s lives is a crucial topic for both the government and academia. We delve into the prerequisites for reducing household financial vulnerability through financial spatial structure, focusing on financial regulation and technology. This can offer policy insights for deepening financial supply-side structural reforms and advancing the establishment of modern financial systems.

## 2. Theoretical analysis and research hypotheses

### 2.1 Literature review

#### 2.1.1 Research on the influencing factors of household financial fragility

Household financial vulnerability refers to the probability of a household encountering financial hardships in the foreseeable future. Academic scholars have conducted substantial research into the factors influencing household financial vulnerability, concentrating on three main aspects: individual traits, familial aspects, and extrinsic factors. Initially, previous investigations have explored how the householder’s gender, age, education and cognitive abilities shape household financial vulnerability. Numerous studies suggest that older householders, those with lesser educational attainment and limited financial knowledge, as well as female householders, tend to face elevated financial vulnerability [6–10]. Moreover, prior research has mainly focused on factors at the familial level, encompassing elements such as household size, assets, income and expenses, etc. [11–13]. The essence of household financial vulnerability suggests that augmenting household income and assets, along with mitigating expenses and liabilities, can alleviate financial vulnerability. Empirical data from multiple nations backs this assertion. For instance, a study by Yusof et al. (2015) [14] analyzing Malaysian urban households revealed that merely 10% could withstand shocks stemming from unemployment, divorce, death, or fluctuations in interest rates or the stock market. If family income is interrupted, over one-fifth of households cannot survive for a minimum of three months. Research by Sdnchez-Martfnez et al. (2016) [15] indicated that the financial crisis caused by the housing bubble burst led to the unemployment shock and decline of disposable household income and increased economic vulnerability in Spain, especially among married and self-employed women with mortgages. Finally, research on the external factors of the family revolves around natural disasters and the macroeconomic environment. Yao and Xu (2018) [16] and Zhang and Liu (2021) [17] found that earthquakes can affect household income, savings and consumption, thereby affecting household financial vulnerability. Few scholars have examined the impact of macro factors such as the supply of financial resources, aging population, and institutional environment on household financial activities [18,19].

#### 2.1.2 Relevant research of “local market effects” and “spatial spillover effects” of finance

The financial spatial structure pertains to the distribution of financial resources across various regions, and a well-balanced financial structure signifies financial progress [5,20]. Competitive advantages, innovations, and economies of scale lead to the concentration of financial resources in specific areas, thereby influencing local economic endeavors. Studying the "local market effects" and "spatial spillover effects" of the financial structure delves deeper into the economic impacts of this agglomeration. According to recent investigations, such as those conducted by Dai et al. (2022) [20] and Tao et al. (2017) [21], the clustering of local financial institutions fosters a more comprehensive regional financial system, which in turn aids market entities in overcoming financing obstacles originated from information opacity, minimizing transaction costs and mitigating risk control challenges posed by geographical distances. Collectively, these impacts are referred to as the local market effects of finance. As urban financial resources diverge and the financial system integrates further, financial agglomeration naturally evolves, leading to the emergence of central ‐ periphery structure of financial centers [22]. In China, regional financial centers are significant hubs for financial activities, with a high density of financial institutions, abundant financial resources, and robust financial service capabilities. Leveraging these strengths, financial agglomeration intensifies, cumulative competitive and innovative advantages emerge, and the scope and sophistication of these financial centers progressively expand [21]. Scholars define the influence of central city financial markets on the economic endeavors of adjacent cities as "financial spillover effects". Financial central cities have both “local market effects” and “spatial spillover effects”. While these two types of effects have heterogeneous effects on market entities, research indicates that both contribute positively to augmenting enterprise export scale [20], bolstering regional innovation capabilities [23] and driving regional economic growth [24].

#### 2.1.3 The effects of financial structure on household financial vulnerability

Regarding the effects of financial structure on household financial vulnerability, current academic researches primarily focus on the influence of financial intermediaries and financial instruments. Firstly, the optimization of the financial structure enhances households’ credit availability, helps intertemporal smoothing and reduce their financial vulnerability [25–27]. In particular, the rapid development of financial technology has significantly improved the efficiency and boundary financial services [28,29]. Furthermore, the optimization of the financial structure provides households with diversified asset allocation options, thereby diversifying household financial risks and reducing vulnerability [30]. Reasonable planning and allocation of insurance products can ensure that households have sufficient financial supports when facing needs at different life stages (such as retirement planning, medical expenses and family entrepreneurship), which helps to develop stable financial expectations [31,32]. Lastly, the increase in the number of various financial institutions can reduce information asymmetry, providing households with abundant financial information resources [33]. The widespread application of technologies, such as the Internet, big data and artificial intelligence, has made financial information more accessible to households. Additionally, through personalized recommendations and intelligent analyses, these technologies help households to obtain the information they need more precisely [34,35].

Currently, academic research on the effects of financial structure on household finance primarily concentrate on the influence of financial intermediaries and financial instruments, such as banks and financing methods, on households. There is less research exploring the subtle relationship between core-periphery financial spatial structure and household activities from the perspective of financial geography, particularly from the angles of financial centers in China’s provincial capitals and local finance. With the in-depth development of China’s urbanization and agglomeration construction, as well as the deployment of high-quality financial advancement, studying and exploring the effects and mechanisms of financial spatial structure on household financial vulnerability, especially how the construction and development of regional financial centers and local financial markets affect household finance, will enhance our understanding of the logical relationship between financial market development and household financial risk prevention. This will also provide a theoretical basis for the future development of China’s financial spatial structure.

### 2.2 Theoretical analysis and research hypotheses

The causes of household financial vulnerability stem from factors such as low household income, insufficient savings, low financial literacy, as well as a lack of effective investment portfolios. With the continuous deepening of household participation in financial markets, both “local market effects” and “spatial spillover effects” of finance can exert influence on household financial vulnerability through various channels. In light of the causes of household financial vulnerability, we will elucidate the impact of financial spatial structure on household financial vulnerability from three key aspects: household entrepreneurship, household asset allocation, and residents’ financial literacy.

#### 2.2.1 Household entrepreneurship

In the context of "business startups and innovation initiatives", the Chinese government has effectively implemented various entrepreneurial support policies aimed at enhancing the external environment for individual businesses and presenting ample entrepreneurial opportunities. The impact of entrepreneurial endeavors on improving household economic status and boosting income has been extensively acknowledged through research [36,37]. However, constrained by insufficient savings, lack of collateral assets, and persistently high interest rates in private lending, many households’ entrepreneurial activities are hindered [38]. The “local market effects” and “spatial spillover effects” of finance can enhance the accessibility of households’ credit resources, contributing to stimulating household entrepreneurial vitality. The specific reasons are that: firstly, the agglomeration of financial resources exhibits the “scale effects”, which can enhance the efficiency of capital operations in the regional financial market [21], shorten the capital turnover cycle, thereby reducing household financing costs and encouraging households to engage in entrepreneurial activities or expand production scale. Secondly, the “competitive effects” of financial resources agglomeration can facilitate collaboration among different financial institutions, thereby promoting financial institutions to launch new products and services such as risk-cybernetics and online lending [39], meeting the financing needs of various households through more convenient services, lower interest rates, longer repayment terms, and so on. Thirdly, the “network effects” of financial resources agglomeration helps reduce information acquisition costs for both financial institutions and households and promotes the transmission and sharing of information [40], enabling financial institutions to identify household financing needs more promptly and accurately and assisting entrepreneurial households in finding suitable loan project. Obtaining financial support means that these households with financial constraints can continue to engage in entrepreneurial activities or expand their businesses [41], which will increase household income and reduce household financial vulnerability.

#### 2.2.2 Household asset allocation

Prudently allocating assets and establishing diversified investment portfolios are crucial for enhancing household abilities to withstand risks and alleviating household financial vulnerability. Particularly, insurance financial products can provide considerable revenue for the household when there are no insurance claims at the policy mature and can also offer insurance coverage several times higher than the premium paid in the event of an insurance incident [42], thereby mitigating the impact of unforeseen events on households. The “local market effects” and “spatial spillover effects” of finance provide more options for household asset allocation [43], allowing residents to access a variety of high-quality insurance financial products. Households can purchase various types of insurance based on their needs and economic conditions, such as education savings insurance, medical insurance, life insurance, loan insurance. By leveraging the dual functions of protection and returns offered by insurance financial products, households can enhance their resistance to risks.

#### 2.2.3 Residents’ financial literacy

Financial literacy refers to the individual’s understanding of basic economic concepts and foundational financial knowledge, as well as the ability to process economic information and make sound financial decisions [44]. It can affect household participation in the stock market [41,45], the purchase of commercial insurance [46], and the availability of formal credit [47], and is considered as an important factor influencing household financial vulnerability [48,49]. Households with higher financial literacy are able to better identify potential financial risks, enabling them to choose suitable financial products and services and helping them avoid unnecessary debt and financial pitfalls. Moreover, households with higher financial literacy are more capable of effective financial planning and decision-making. Through managing household budgets, planning for retirement and children’s education funds, and optimizing investment portfolios, they can reduce their risk exposure and better cope with unpredictable external shocks. Conversely, due to a lack of essential financial knowledge and an inability to accurately assess the true risks of credit, investment tools, or banking-related transactions, households lacking financial literacy are prone to issues such as excessive debt, credit defaults, and difficulties in repayment, thereby increasing household financial vulnerability.

The "local market effects" and "spatial spillover effects" inherent in the financial spatial structure play a significant role in enhancing residents’ financial literacy. In regions with a concentration of financial resources, the presence of multiple active financial institutions increases the transparency of financial information, facilitates the rapid dissemination of financial knowledge [23], and establishes favorable conditions for elevating the financial literacy of the local populace. Based on these advantages, residents will learn more knowledge about investment strategies and risk management techniques, allowing them to participate in financial activities more actively. On the other hand, with the continuous innovation of financial products and the intensification of market competition, the importance of financial literacy for household wealth management is increasingly prominent, indirectly reinforcing residents’ subjective willingness to enhance their own financial literacy.

Based on the above, the following hypotheses are proposed:

**H1**. The “local market effects” and “spatial spillover effects” inherent in financial spatial structure contribute to alleviating household financial vulnerability.
**H1a.** The mechanisms by which financial spatial structure influences household financial vulnerability include promoting household entrepreneurship, optimizing household asset allocation, and enhancing residents’ financial literacy.

### 2.3 The moderating effect of financial regulation and financial technology

Existing studies indicate that there is a significant regional disparity in the supply of financial resources in China, resulting in asymmetric effects of “local market effects” and “spatial spillover effects” on the real economy [20,21,23]. At the same time, with the continuous strengthening of financial regulation and the rapid development of digital technology, the financial environment faced by households is also undergoing constant changes. In order to further explore how to strengthen the effect of financial spatial structure on household financial vulnerability, we introduce regional financial regulation and financial technology development into the analytical framework.

#### 2.3.1 The moderating effect of financial regulation

Effective financial regulatory mechanisms serve as the cornerstone for the healthy growth of financial markets. In recent years, the Chinese government has implemented a range of policies to enhance financial supervision comprehensively, aiming to expedite the journey towards becoming a financially resilient nation and fostering high-quality financial progress. Notably, in 2023, the government enacted the Regulations on the Supervision and Administration of Non-bank Payment Institutions, marking the first administrative regulation in China dedicated to overseeing non-bank payment institutions. These regulations prioritize risk prevention and resolution, protect the legitimate rights and interests of users, emphasize comprehensive supervision throughout the entire business cycle, and mitigate risks like business deviation, fund misappropriation and data leakage. This legislative framework provides regulatory basis for promoting compliant operation of payment institutions, purifies the market environment, and offers clear guidance and effective oversight for all parties in the industry [50]. This paper argues that financial regulation can strengthen the “local market effects” and “spatial spillover effects” of finance. Regulatory agencies, by establishing uniform rules and standards and vigorously combatting illegal activities such as insider trading and market manipulation, can maintain a healthy market order, regulate the behavior of financial institutions, and protect household investors from undue exploitation and deception by financial institutions. At the same time, information sharing and disclosure mechanisms will enhance the accuracy, transparency, and suitability of information disclosure in the business process of financial institutions, contributing to households making reasonable investment decisions and risk management, thereby improving the effectiveness of household asset portfolios. Conversely, the lack of effective financial regulation is capable of increasing market instability and unfairness, leading to a crisis in the entire financial system [51]. For example, lower entry thresholds can disrupt the order of the financial markets and result in the financial market being filled with inexperienced and unskilled financial institutions and practitioners, that is, there is a phenomenon of “bad money driving out good”. At that time, improper sales and fraudulent activities by financial institutions will occur frequently, causing losses to household investors.

Based on this, we propose the following hypothesis:

**H2**. Financial regulation can strengthen the inhibitory effect of the local financial market on household financial vulnerability.

#### 2.3.2 The moderating effect of financial technology

The rapid development of financial technology has brought about a profound revolution in the financial industry. Leveraging technologies such as big data, cloud computing, and artificial intelligence, financial institutions have achieved more efficient financial operations and more precise risk control [52], thus further strengthening the “local market effects” and “spatial spillover effects” of finance. On the one hand, financial institutions can use digital technology to assess household credit risk and track their fund usage more accurately. This helps reduce information asymmetry between the demanders and suppliers of funds, thereby enhancing the accessibility of credit resources for households [53]. On the other hand, households can choose financial services and products from a broader range, optimize their investment portfolios, and better diversify risks, thereby reducing household financial vulnerability. In addition, the continuous advancement of financial technology integration and innovation provides residents with faster and more convenient financial services such as payments, transfers, and loans, enabling households to manage their finances more flexibly, thereby reducing household financial vulnerability. Therefore, we propose the following hypothesis:

**H3**. Financial technology can strengthen the inhibitory effect of the local financial market on household financial vulnerability.

## 3 The empirical strategy

### 3.1 Data sources

This paper utilizes two primary datasets: urban financial spatial structure data and household financial data. The former was sourced from the China Urban Statistical Yearbook, China Statistical Yearbook, and the Economy Prediction System (EPS) data platform (https://www.epsnet.com.cn). Meanwhile, the latter originates from the Chinese Family Panel Studies, a comprehensive survey organized and executed by Peking University (http://www.isss.pku.edu.cn/cfps/sjzx/gksj/index.htm). The Chinese Family Panel Studies offers two significant strengths. Firstly, its sample is highly representative and extensive, encompassing a wide range of households. Since 2010, the survey has been successfully administered in six rounds, reaching a total of 19,000 household samples in 2020. These samples span 919 districts and counties across 31 provinces in China, bolstering the empirical analysis presented in this paper. Secondly, the survey’s rich content, embracing an abundance of information related to household financial assets and liabilities, facilitates a comprehensive understanding of household financial activities. For our analysis, we have carefully selected publicly available data spanning from 2012 to 2020 as the initial sample. After rigorous data cleaning, excluding observations with severe missing data, our final dataset comprises 25,367 observations from 6,822 households, spread across 98 cities in China. This robust dataset forms the foundation for our in-depth exploration of the topic at hand.

### 3.2 Model

Firstly, to identify the impact of financial spatial structure on household financial vulnerability, referring to [18], we set the following Probit model:

$$Prob(Fragile_{ict}=1|X_{it})=\alpha_{0}+\alpha_{1}Finance_{ict}+\alpha X_{it}+\lambda_{t}+\xi_{ict}$$ (1)

where, subscripts *i*, *c*, and *t*, represents household, city, and year, respectively. *Fragile* is household financial vulnerability, as a binary variable. *Finance* represents urban financial spatial structure, including both the “local market effects” and “spatial spillover effects” of finance. Based on the previous analysis, if *α*_1_ is significantly negative, indicating that financial spatial structure will reduce household financial vulnerability, namely, confirming **H1**. *X*_*it*_ represents a set of control variables, *λ_t_* denotes time fixed effects, and *ξ_ict_* represents the random disturbance term.

Secondly, to examine the mechanism that financial spatial structure affects household financial vulnerability, the following model is set:

$$Prob(Fragile_{ict}=1|X_{it})=\beta_{0}+\beta_{1}Mech_{it}+\beta_{2}Mech_{it}*Finance_{ict}+\beta X_{it}+\lambda_{t}+\xi_{ict}$$ (2)

where, *Mech* indicates the mechanism variables, including household entrepreneurship, household asset allocation, and residents’ financial literacy. If *β*_1_ and *β*_2_ are all significantly negative, verifying **H1a**. The meaning of other variables is the same as Eq (1).

Finally, to explore whether financial regulation and financial technology can strengthen the inhibitory effect of financial spatial structure on household financial vulnerability, we introduce the interaction terms between regional financial regulatory, financial technology and financial spatial structure (Finance*Regulation and Finance*Fintec) based on Eq (1). The specific model is as follows:

$$\begin{array}{l} Prob(Fragile_{ict}=1|X_{it})=\theta_{0}+\theta_{1}Finance_{ict}+\theta_{2}Finance_{ict}*Regulation_{ict}\\ +\theta X_{it}+\lambda_{t}+\xi_{ict} \end{array}$$ (3)

$$\begin{array}{l} Prob(Fragile_{ict}=1|X_{it})=\theta_{0}+\theta_{1}Finance_{ict}+\theta_{2}Finance_{ict}*Fintec_{ict}\\ +\theta X_{it}+\lambda_{t}+\xi_{ict} \end{array}$$ (4)

If the estimated coefficient *θ*_2_ is significantly negative, it indicates that financial regulation and financial technology can strengthen the inhibitory effect of financial spatial structure on household financial vulnerability, confirming **H2** and **H3**. The meanings of other variables are the same as in Eq (1).

### 3.3 Variables

#### 3.3.1 The explained variable: Household financial vulnerability (*Fragile*)

Referring to [18], we adopt two methods to measure household financial vulnerability. One method is based on the “financial deposit” indicator proposed by [54] to measure household financial vulnerability (*Fragile_Deposit*), that is, by calculating whether household income and risk-free assets can cover household expected and unexpected expenditures. The specific calculation method is as follows:

$$Fragile_{it}=FM_{it}^{A}+LA_{it}-UE_{it}$$ (5)

where, $FM_{it}^{A}$ indicates the expected financial deposit for households, which is equal to the total family income minus living expenses, mortgage, and related interest. *LA*_*it*_ indicates risk-free assets, mainly including the family’s cash and deposits. *UE*_*it*_ refers to unexpected expenditures, measured by the approach of [18], which includes the unplanned education and medical expenditures of households. If *Fragile*_*it*_ is less than zero, it is indicated that the family has household financial vulnerability and the value of *Fragile*_*Deposit* is 1; otherwise, *Fragile*_*Deposit* takes the value of 0.

The second method involves using the “emergency savings” indicator proposed by [55] to measure household financial vulnerability (*Fragile*_*Saving*). If household savings are insufficient to cover three months of daily expenses and the household debt-to-income ratio is greater than 30%, the household is considered financial vulnerability, and the value of *Fragile*_*Saving* is 1; otherwise, *Fragile*_*Saving* takes the value of 0.

#### 3.3.2 Explanatory variable: Financial spatial structure (*Finance*)

Referring to [20,21], we examine the financial spatial structure from “local market effects” and “spatial spillover effects”. The “local market effects” of finance is measured by the natural logarithm of the financial geographic density of the city, and the model is as follows:

$$Lnlocal_{ict}=\log(Loan_{ict}/Area_{ic})$$ (6)

where, subscripts *i*, *c*, and *t*, represents household, city, and year, respectively. *Lnlocal* is the “local market effects” of finance. *Loan* is the financial activity scale in the city, measured by the loan balance of financial institutions at the end of the year. *Area* is the built-up area of the city.

Referring to [20,21], the geographical distance between prefecture-level cities and provincial capital cities is incorporated into the calculation formula for financial geographical density to measure the financial spatial spillover effects, and the model is set as follows:

$$Lncentre_{ict}=\log(Loan_{iht}/Distance_{ch}*Area_{h})$$ (7)

where, *h* represents the provincial capital city. *Lncentre* represents the “spatial spillover effects” of finance, that is, the spillover degree of regional financial centers to peripheral cities. *Loan*_*ih*_ is the scale of provincial capital cities’ financial activities. *Distance*_*ch*_ indicates the spatial distance between prefecture-level cities *c* and provincial capital city *h*. *Area*_*h*_ represents the built-up area of the provincial capital city *h*.

#### 3.3.3 Mechanism variables

*Household entrepreneurship (Dum_Entrep)*. In the CFPS questionnaire, respondents were asked, “In the past 12 months, have any members of your household engaged in individual business or started a private enterprise?” Based on the response to this question, we defined the household entrepreneurship variable. If the respondent answered “yes”, the value of *Dum_Entrep* is 1; otherwise, *Dum_Entrep* equals 0.

*Household asset allocation (Dum_Insurance)*. We measure household asset allocation based on whether the household has purchased commercial insurance. In the CFPS questionnaire, respondents were asked, “In the past 12 months, how much did your family spend on purchasing commercial insurance such as commercial medical insurance, property insurance, commercial life insurance, etc.?” Based on the answer to this question, we define household asset allocation variable. If the family’s expenditure on commercial insurance is greater than zero, the value of *Dum_Insurance* is 1; otherwise, *Dum_Insurance* equals 0.

*Residents’ financial literacy (Literacy)*. The CFPS questionnaire contains a large number of questions about residents’ financial knowledge, investment preferences, and investment behaviors. According to [56], the entropy weight method is employed to comprehensively assess the residents’ financial literacy based on their financial knowledge, financial attitude, and financial behavior. The specific indicator system and weight is shown in **Table 1**.

**Table 1:** The evaluation system of residents’ financial literacy.

Financial Literacy	Indicator and corresponding questions in CFPS	Weight
Financial Knowledge (L1, 0.6423)	“The one-year fixed interest rate of banks”-What do you estimate the current interest rate for one-year fixed deposits in banks?	0.0760
	“Maturity amount of deposit”-Assuming you have a one-year fixed deposit of 10000 yuan with an annual interest rate of 3%, if you do not withdraw it in advance, how much money will you have after the deposit matures?	0.0999
	“Renewal amount of deposit”-How much money is there in the account after the deposit in the previous question expires and is deposited for another year with a fixed term at the same interest rate?	0.0852
	“Purchasing power of currency”-If your bank deposit account has an annual interest rate of 3% and an inflation rate of 5% per year. So, how much can you buy with the money from this account in one year?	0.0676
	“Value comparison”-Assuming that Zhang San inherits 100000 yuan today, and Li Si will inherit 100000 yuan in 3 years. So, which of them has a higher inheritance value?	0.1149
	“Investment risk”-In general, high-yield investments carry high risks.	0.1242
	“Stock investment risk”-In general, investing in a single stock carries less risk than investing in equity funds.	0.0551
	“Decision making Bank”-Which of the following banks has the function of formulating and implementing monetary policy?	0.0767
	“Risks of investing products”-Generally speaking, which of the following assets has the highest risk in terms of investment product risk?	0.0576
	“Meaning of purchasing stocks”	0.0637
	“Fund Description”	0.0490
	“The description of financial product”	0.0785
	“The description of stock market functions”	0.0516
Financial Behavior (L2, 0.2536)	“Shopping affordability”-When I make purchases, I consider my own affordability	0.0982
	“Pay bills on time”-I pay bills on time (such as water, electricity, gas bills, credit cards, etc.)	0.0549
	“Pay attention to financial situation”-I pay close attention to my financial situation, such as frequently checking my bank account and income and expenditure status.	0.0876
	“Make long-term financial planning”-I make long-term financial plans.	0.1273
	“The management Financial revenue and expenditure”-I manage my own financial income and expenses, as well as household financial income and expenses.	0.0837
	“Consumption model”-I live within my means, which means I will consume based on my income.	0.0578
	“Choose the financial product model”-When choosing the financial product model, I collect product information and compare various products.	0.1319
	“Loan usage”-I will use borrowing and lending to maintain a balance of income and expenditure.	0.2066
	“Keep the account”-I have a habit of keeping accounts and can record all income and expenses.	0.1520
Financial Attitude (L3, 0.1041)	“Subjective feelings”-My subjective feeling is that spending money is more satisfying than saving it.	0.3726
	“Subjective inclination”-I am more inclined to live in the present and not consider future things with a subjective tendency.	0.3146
	“Money view”-I believe that money is meant to be spent, and if you have money, you have to spend it.	0.3128

#### 3.3.4 Moderating variables

*Financial regulation (Regulation)*. Referring to [50], we use the ratio of governments’ financial regulatory expenditure to the added value of the financial industry as a proxy variable for the intensity of regional financial regulation.

*Financial technology (Fintec)*. Referring to the research of [53], we use the China Digital Inclusive Finance Index released by Peking University as a proxy variable for the level of regional financial technology development.

#### 3.3.5 Control variables

This paper incorporates control variables at both the householder and family levels. At the householder level, control variables include the householder’s age (*Age*) and its squared term (*Agesq*), gender (*Gender*), party (*Dum_Party*), college (*Dum_Edu*). At the family level, control variables consist of the proportion of children under 6 years old (*Young*), the proportion of people over 60 years old (*Old*), and family population (*Family Size*). Descriptive statistics of the main variables are presented in **Table 2**.

**Table 2:** The descriptive statistics of main variables.

Variable	Obs	Mean	Std.	Min	Max
*Fragile_Deposit*	25367	0.393	0.488	0	1
*Fragile_Saving*	25367	0.513	0.500	0	
*Lnlocal*	25367	7.415	1.725	4.289	11.720
*Lncentre*	20212	4.069	1.072	1.305	7.257
*Dum_Entrep*	25367	0.095	0.293	0	1
*Dum_Insurance*	17943	0.810	0.392	0	1
*Literacy*	6494	1.129	0.323	0.348	2.713
*Age*	25367	50.54	13.59	16	95
*Gender*	25367	0.570	0.495	0	1
*Dum_Edu*	25367	0.021	0.145	0	1
*Dum_Party*	25367	0.072	0.258	0	1
*Dum_Married*	25367	0.699	0.459	0	1
*Young*	25367	0.029	0.091	0	0.750
*Old*	25367	0.262	0.354	0	1
*Family Size*	25367	2.994	1.397	1	14

## 4 Empirical results

### 4.1 Benchmark regression

**Table 3** reports the estimation results of Eq (1). Columns (1) and (2) are the results using *Fragile_Deposit* as the explained variable. The coefficients of *Lnlocal* and *Lncentre* are -0.120 and -0.092, respectively, and significant at the 1% level, indicating that “local market effects” and “spatial spillover effects” of finance is conducive to alleviating household financial vulnerability. When the level of local financial development (“local market effects” of finance) and the construction of regional financial center (“spatial spillover effects” of finance) increase by 1%, the degree of household financial vulnerability will decrease by 0.12 and 0.09, respectively. We obtain the similar results using *Fragile_Saving* as the explained variable in columns (3) and (4), and the estimated coefficients of *Lnlocal* and *Lncentre* still significantly negative. Thus, **H1** has been preliminarily validated.

**Table 3:** Financial spatial structure and household financial vulnerability.

	(1)	(2)	(3)	(4)
	*Fragile_Deposit*	*Fragile_Deposit*	*Fragile_Saving*	*Fragile_Saving*
*Lnlocal*	-0.120***		-0.138***	
	(0.015)		(0.013)	
*Lncentre*		-0.092***		-0.102***
		(0.020)		(0.028)
*Age*	0.007	0.011**	0.030***	0.029***
	(0.005)	(0.005)	(0.005)	(0.006)
*Agesq*	-0.000**	-0.000**	-0.000***	-0.000***
	(0.000)	(0.000)	(0.000)	(0.000)
*Gender*	-0.076***	-0.057**	-0.111***	-0.098***
	(0.022)	(0.026)	(0.022)	(0.028)
*Dum_Edu*	-0.148**	-0.188**	-0.175***	-0.245***
	(0.062)	(0.079)	(0.057)	(0.072)
*Dum_Party*	-0.127***	-0.102**	-0.228***	-0.186***
	(0.041)	(0.046)	(0.039)	(0.048)
*Dum_Married*	-0.107***	-0.082**	-0.140***	-0.137***
	(0.037)	(0.034)	(0.035)	(0.033)
*Young*	0.600***	0.621***	0.184*	0.238**
	(0.103)	(0.116)	(0.095)	(0.108)
*Old*	0.261***	0.309***	0.050	0.047
	(0.046)	(0.047)	(0.036)	(0.043)
*Family Size*	-0.047***	-0.045***	0.005	0.009
	(0.007)	(0.007)	(0.009)	(0.010)
Constant	0.504***	-0.066	0.223	-0.307*
	(0.162)	(0.141)	(0.141)	(0.176)
Year Fix Effect	Yes	Yes	Yes	Yes
Observations	25367	20212	25367	20212

Note: ***, ** and * indicate significance at the 1%, 5% and 10% levels, respectively. The clustered standard error at the city level is reported in parentheses.

### 4.2 Robustness test and endogeneity analysis

First, to isolate the impact of regional characteristics on household financial vulnerability, we introduce additional variables into Eq (1): the level of urban economic development (GDP, measured by the natural logarithm of per capita GDP), urban transportation conditions (Pass, measured by the natural logarithm of regional passenger volume), and urban communication level (Tele, measured by the natural logarithm of regional postal and telecommunications business volume). The estimation results incorporating these variables are presented in columns (1) to (4) of Table 4. Additionally, compared to ordinary prefecture-level cities, municipalities directly under the central government and provincial capitals have higher functional orientation and stronger resource endowment. To eliminate this interference, we only use the samples consisting of prefecture-level cities for regression when examining the impact of the “local market effects” of finance on household financial vulnerability. The estimation results are shown in columns (5) and (6) of **Table 4**. In the above regression, the estimated coefficients of *Lnlocal* and *Lncentre* are both significantly negative, which are consistent with the results of the baseline regression.

**Table 4:** Robustness test: Control for other influencing factors.

	(1)	(2)	(3)	(4)	(5)	(6)
	*Fragile_Deposit*	*Fragile_Deposit*	*Fragile_Saving*	*Fragile_Saving*	*Fragile_Deposit*	*Fragile_Saving*
*Lnlocal*	-0.123***		-0.136***		-0.112***	-0.133***
	(0.024)		(0.028)		(0.020)	(0.025)
*Lncentre*		-0.065***		-0.063**		
		(0.021)		(0.028)		
*GDP*	0.041	-0.092**	-0.014	-0.168***		
	(0.043)	(0.045)	(0.062)	(0.059)		
*Pass*	-0.043*	-0.050	-0.057*	-0.077*		
	(0.024)	(0.031)	(0.033)	(0.040)		
*Tele*	0.008	-0.011	0.033	0.006		
	(0.028)	(0.028)	(0.031)	(0.032)		
Controls	Yes	Yes	Yes	Yes	Yes	Yes
Year FE	Yes	Yes	Yes	Yes	Yes	Yes
N	25367	20212	25367	20212	20212	20212

Note: ***, ** and * indicate significance at the 1%, 5% and 10% levels, respectively. The clustered standard error at the city level is reported in parentheses.

Secondly, we address endogeneity issues caused by unobservable factors at the individual, family, and city levels, which may influence household financial vulnerability, drive the demand for financial resources, and ultimately affect regional financial development. To mitigate this impact, we restrict the sample range, following the approach outlined in [53]. Firstly, individuals with higher capabilities are more likely to grasp financial knowledge and manage household wealth effectively, which can lead to a reduction in household financial vulnerability. To alleviate endogeneity caused by individual capabilities, we restrict the sample to householders with lower capabilities. The score of the householder’s intelligence level is used as the proxy for householder’s capabilities and the estimation results are presented in columns (1) and (2) of **Table 5**. Secondly, household social relationships or family environment may affect household financial activities. Typically, wealthy families have relatively abundant social relationships. We use household income as the proxy variable for household social resources and restrict the sample to low-income families to mitigate the endogeneity brought by unobservable factors at the family level. The estimation results are presented in columns (3) and (4) of **Table 5**. Finally, factors such as urban entrepreneurial atmosphere, employment environment, and residents’ attitudes towards venture capital will affect household financial participation. To mitigate the endogeneity brought by unobservable factors at the regional level, we restrict the sample to regions with a lower level of economic development and the estimation results are presented in columns (5) and (6) of **Table 5**. After considering the impact of unobservable factors at the individual, family, and city levels on the estimation results, we find that the estimated coefficients of *Lnlocal* and *Lncentre* are both significantly negative, indicating that the benchmark conclusion is robust.

**Table 5:** Robustness test: Considering the endogeneity problems caused by reversal casualty.

	(1)	(2)	(3)	(4)	(5)	(6)
	Low individual capabilities		Low household wealth		Regions with low economic development	
*Lnlocal*	-0.116***		-0.144***		-0.112***	
	(0.019)		(0.015)		(0.015)	
*Lncentre*		-0.081***		-0.087***		-0.085***
		(0.024)		(0.025)		(0.023)
Controls	Yes	Yes	Yes	Yes	Yes	Yes
Year FE	Yes	Yes	Yes	Yes	Yes	Yes
N	11056	8672	12615	9991	13701	10806

Note: ***, ** and * indicate significance at the 1%, 5% and 10% levels, respectively. The clustered standard error at the city level is reported in parentheses.

Thirdly, to identify the causal effect of financial spatial structure on household financial vulnerability, we employ the instrument variable (IV) method. This approach is necessary due to the potential existence of omitted variables and the possibility of other factors simultaneously affecting both household financial vulnerability and the financial spatial structure. Specifically, we use the lagged explanatory variable as an instrumental variable and conduct a two-stage least squares (2SLS) estimation. **Table 6** reports the results. The results of Kleibergen-Paap rm LM statistic and Kleibergen-Paap Wald F statistic reject the null hypothesis of under-identification and the weak instrumental variables, indicating that the IVs chosen in this paper are reasonable. The results of the second stage indicate that, after considering the endogeneity issue, the impact of the “local market effects” and “spatial spillover effects” of financial spatial structure on household financial vulnerability remains significantly negative, confirming the robustness of the benchmark conclusion.

**Table 6:** Robustness test: IV method.

	(1)	(2)	(3)	(4)
	*Lnlocal*	*Fragile_Deposit*	*Lncentre*	*Fragile_Deposit*
*IV*	0.960***		0.979***	
	(0.002)		(0.006)	
*Lnlocal*		-0.048***		
		(0.003)		
*Lncentre*				-0.035***
				(0.005)
Controls	Yes	Yes	Yes	Yes
Year FE	Yes	Yes	Yes	Yes
Kleibergen-Paap rk LM statistic	1764.215		1675.870	
Kleibergen-Paap rk Wald statistic	15000		30000	
N	15266	15266	12196	12196

Note: ***, ** and * indicate significance at the 1%, 5% and 10% levels, respectively. The clustered standard error at the city level is reported in parentheses.

Finally, using Logit model to re-examine. We relax the assumption that household financial vulnerability obeys a normal distribution and use the Logit model for estimation. The results are presented in **Table 7**. The estimated coefficients for *Lnlocal* and *Lncentre* are both significantly negative. Compared to the baseline regression results, the absolute values of the estimated coefficients have increased, further confirming the benchmark conclusion once again.

**Table 7:** Robustness test: Using Logit model.

	(1)	(2)	(3)	(4)
	*Fragile_Deposit*	*Fragile_Deposit*	*Fragile_Saving*	*Fragile_Saving*
*Lnlocal*	-0.195***		-0.223***	
	(0.025)		(0.021)	
*Lncentre*		-0.149***		-0.163***
		(0.032)		(0.045)
Controls	Yes	Yes	Yes	Yes
Year FE	Yes	Yes	Yes	Yes
N	25367	20212	25367	20212

Note: ***, ** and * indicate significance at the 1%, 5% and 10% levels, respectively. The clustered standard error at the city level is reported in parentheses.

## 5 Mechanism test

### 5.1 Promote household entrepreneurship

Columns (1) to (4) of **Table 8** reports the estimation results using household entrepreneurial choice (*Dum_Entrep*) as the mechanism variable. The estimated coefficient of *Dum_Entrep* is significantly negative at the 1% level, and the estimated coefficient of *Lnlocal × Dum_Entrep* is also significantly negative at the 1% level. Although entrepreneurial activities come with higher risks, they can improve the economic condition of households and increase family income, playing a positive role in reducing household financial vulnerability. The “local market effects” of finance can broaden entrepreneurs’ financing channels, enhance the accessibility of credit resources for households, and better meet the financing needs of entrepreneurial families. The estimated coefficient of *Lncentre×Dum_Entrep* is negative but did not pass the significance test. This could be attributed to the preference of borrowers to obtain loans from banks with the closest geographical distance [57]. Specifically, entrepreneurial families that lack of mortgaged assets, benefiting from the construction of a regional financial center may be more challenging. Therefore, the logic that “spatial spillover effects” of finance reduce household financial vulnerability by stimulating entrepreneurial activities was not supported by empirical evidence.

Although entrepreneurship offers families expanded opportunities for income and wealth accumulation, it also poses significant risks and challenges. Families lacking the prerequisites for successful entrepreneurship, such as adequate skills, knowledge, experience and the ability to adapt to market fluctuations and adjust business strategies, face a high risk of failure. In such cases, entrepreneurial ventures not only fail to augment household income but may also exacerbate financial hardships. To assess the impact of the quality of household entrepreneurship, we categorize whether the entrepreneurial endeavors of the sampled families constitute high-quality entrepreneurship. Referring to Tian et al. (2023) [53], we define high-quality entrepreneurship (*Dum_High entrance*) from three criteria. One is that household entrepreneurship lasts for more than two years. The second is that the innovation level of the industry which the sample must surpass the average, as determined by data from the "China Urban and Industrial Innovation Power Report 2017" published by Fudan University and the Yicai Research Institute. The third is that the householders have completed high school education. If any of the above conditions are met, *Dum_High* entrance is assigned a value of 1; otherwise, it is 0. The regression results are shown in columns (5) to (8) of **Table 8**. The estimated coefficient of *Dum_High entrep* and *Lnlocal×Dum_High entrep* are significantly negative at the 1% level, while the coefficient of *Lncentre×Dum_Entrep* still has not pass the significance test. When compared to the results in columns (1) to (4), it becomes evident that the absolute values of the coefficients for *Dum_High entrep* and *Lnlocal×Dum_High entrep* are larger, indicating that high-quality entrepreneurial activities have a more pronounced effect in reducing household financial vulnerability.

**Table 8:** Mechanism test: Promote household entrepreneurship.

	(1)	(2)	(3)	(4)	(5)	(6)	(7)	(8)
	*Fragile_Deposit*	*Fragile_Deposit*	*Fragile_Saving*	*Fragile_Saving*	*Fragile_Deposit*	*Fragile_Deposit*	*Fragile_Saving*	*Fragile_Saving*
*Dum_Entrep*	-0.129***	-0.144***	-0.162***	-0.182***				
	(0.046)	(0.047)	(0.048)	(0.051)				
*Lnlocal×Dum_Entrep*	-0.047***		-0.050***					
	(0.015)		(0.014)					
*Lncentre×Dum_Entrep*		-0.015		-0.016				
		(0.068)		(0.079)				
*Dum_High entrep*					-0.156***	-0.232***	-0.197***	-0.235***
					(0.015)	(0.021)	(0.064)	(0.086)
*Lnlocal×Dum_High entrep*					-0.142***		-0.110**	
					(0.048)		(0.051)	
*Lncentre×Dum_High entrep*						-0.018		-0.082
						(0.093)		(0.111)
Controls	Yes	Yes	Yes	Yes	Yes	Yes	Yes	Yes
Year FE	Yes	Yes	Yes	Yes	Yes	Yes	Yes	Yes
N	25367	20212	25367	20212	25367	20212	25367	20212

Note: ***, ** and * indicate significance at the 1%, 5% and 10% levels, respectively. The clustered standard error at the city level is reported in parentheses.

### 5.2 Optimize household asset allocation

**Table 9** report the estimation results using household asset allocation as the mechanism variable. In the columns (1) to (4), the estimated coefficients of *Dum_Insurance* are all significantly negative at the 1% level and the coefficients of *Lnlocal×Dum_Insurance* and *Lncentre×Dum_Insurance* are also significantly negative at the 1% level, indicating that “local market effects” and “spatial spillover effects” of finance provide residents with diversified choices for asset allocation. Households can enhance financial security and stability by purchasing insurance financial products to cope with unexpected events, illnesses, property losses, and other risks, thereby reducing the potential significant economic losses. The results are consistent with the conclusion of [42].

**Table 9:** Mechanism test: Optimize household asset allocation.

	(1)	(2)	(3)	(4)	(5)	(6)	(7)	(8)
	*Fragile_Deposit*	*Fragile_Deposit*	*Fragile_Saving*	*Fragile_Saving*	*Fragile_Deposit*	*Fragile_Deposit*	*Fragile_Saving*	*Fragile_Saving*
*Dum_Insurance*	-0.154***	-0.107***	-0.193***	-0.150***				
	(0.027)	(0.029)	(0.029)	(0.031)				
*Llocalfin×Dum_Insurance*	-0.191***		-0.222***					
	(0.023)		(0.028)					
*Lcentrefin×Dum_Insurance*		-0.109***		-0.043***				
		(0.021)		(0.017)				
*Dum_Stock*					0.149***	0.152***	0.202***	0.218***
					(0.019)	(0.021)	(0.014)	(0.023)
*Llocalfin×Dum_Stock*					-0.042***		-0.045***	
					(0.005)		(0.005)	
*Lcentrefin×Dum_Stock*						-0.018***		-0.005*
						(0.004)		(0.003)
Controls	Yes	Yes	Yes	Yes	Yes	Yes	Yes	Yes
Year FE	Yes	Yes	Yes	Yes	Yes	Yes	Yes	Yes
N	17943	18301	17943	18301	14871	14876	14871	14876

Note: ***, ** and * indicate significance at the 1%, 5% and 10% levels, respectively. The clustered standard error at the city level is reported in parentheses.

As a comparison, we also use whether the household purchases stocks (*Dum_Stock*) to measure household asset allocation, and the results are shown in columns (5) to (8). The estimated coefficients of *Dum_Stock* are all significantly positive at the 1% level, indicating that households investing in stocks will aggravate household financial vulnerability, which is align with the reality. The estimated coefficients for *Lnlocal×Dum_Stock* and *Lncentre×Dum_Stock* are both significantly negative, indicating that the “local market effects” and “spatial spillover effects” of finance mitigate the negative impact of risky financial assets on household financial vulnerability. Whether it is local financial market development or the construction of regional financial centers, the development of financial markets can expand household financial knowledge reserves and improve their ability to interpret market information. This enables households to make more reasonable investment choices, thereby enhancing the stability of household finances.

### 5.3 Improve residents’ financial literacy

**Table 10** reports the regression results using residents’ financial literacy as the mechanism variable. The coefficients of *Literacy* are all significantly negative at the 1% level, and the estimated coefficients of *Lnlocal×Literacy* and *Lncentre×Literacy* are both significantly negative at the 1% level, suggesting that the “local market effects” and “spatial spillover effects” of finance can reduce household financial vulnerability by improving residents’ financial literacy. In summary, **H1a** has been verified.

**Table 10:** Mechanism test: Improve financial literacy.

	(1)	(2)	(3)	(4)
	*Fragile_Deposit*	*Fragile_Deposit*	*Fragile_Saving*	*Fragile_Saving*
*Literacy*	-0.367***	-0.328***	-0.592***	-0.620***
	(0.085)	(0.096)	(0.079)	(0.083)
*Lnlocal×Literacy*	-0.193***		-0.174***	
	(0.028)		(0.026)	
*Lncentre×Literacy*		-0.106***		-0.062***
		(0.036)		(0.027)
Controls	Yes	Yes	Yes	Yes
Year FE	Yes	Yes	Yes	Yes
N	6494	4921	6494	4921

Note: ***, ** and * indicate significance at the 1%, 5% and 10% levels, respectively. The clustered standard error at the city level is reported in parentheses.

## 6 Moderating effect test

### 6.1 The moderating effect of financial regulation

**Table 11** reports the results of the moderating effect of financial regulation. The coefficients of *Lnlocal×Regulation* and *Lncentre×Regulation* are all significantly negative, indicating that financial regulation can intensify the inhibitory effect of financial spatial structure on household financial vulnerability. Financial regulators, by establishing rules and standards and regulating the operational activities of financial market participants, contribute to increasing market transparency and compliance, thus ensuring the stability of the financial system. This can prevent monopolies, encourage more financial institutions to enter the market and amplify the inhibitory effect of financial “local market effects” and “spatial spillover effects” on household financial activities. Additionally, the stable financial market can prevent fraudulent activities, protect household interests, and avoid undue harm to residents from fraud and market manipulation, thereby indirectly alleviating household financial vulnerability. **H2** is supported by empirical evidence.

**Table 11:** Moderating effect test of financial regulation.

	(1)	(2)	(3)	(4)
	*Fragile_Deposit*	*Fragile_Deposit*	*Fragile_Saving*	*Fragile_Saving*
*Lnlocal*	-0.111***		-0.137***	
	(0.015)		(0.013)	
*Lnlocal×Regulation*	-0.071**		-0.025***	
	(0.034)		(0.009)	
*Lncentre*		-0.062***		-0.072***
		(0.020)		(0.029)
*Lncentre×Regulation*		-0.035***		-0.017**
		(0.010)		(0.009)
Controls	Yes	Yes	Yes	Yes
Year FE	Yes	Yes	Yes	Yes
N	25367	21894	25367	21894

*Note*: ***, ** and * indicate significance at the 1%, 5% and 10% levels, respectively. *The clustered standard error at the city level is reported in parentheses*.

### 6.2 The moderating effect of financial technology

**Table 12** presents the results of the moderating effect of financial technology. In column (1), the estimated coefficient of *Lnlocal×Fintec* is significantly negative, indicating that the development of financial technology can strengthen the inhibitory effect of the local financial market on household financial vulnerability. Financial technology is a beneficial complement to traditional financial services [58] and significantly improve the quality and efficiency of financial services. From the perspective of financial institutions, banks can leverage big data platforms to assess credit risks and track the financial activities of households, thus alleviating information asymmetry between the demand and supply of funds and enhancing the accessibility of credit resources for households [53]. From the perspective of households, financial technology expands the scope of financial services, reduces the cost for households to access financial services, and enables households to participate in financial activities more actively. Through smart devices and electronic accounts, residents can timely and accurately access their payment, savings, investment, and loan records, helping improve the scientific and rational of personal financial planning, thereby making household finances more robust.

**Table 12:** Moderating effect test of financial technology.

	(1)	(2)	(3)	(4)	(5)	(6)	(7)
*Lnlocal*	-0.110***						
	(0.012)						
*Lnlocal×Fintec*	-0.037***						
	(0.011)						
*Lncentre*		-0.054***	-0.054***	-0.054***	-0.057***	-0.055***	-0.061***
		(0.019)	(0.019)	(0.019)	(0.021)	(0.019)	(0.020)
*Lncentre×Fintec*		-0.010					
		(0.026)					
*Lncentre×Payment*			-0.030**				
			(0.015)				
*Lncentre×Credit*				-0.001			
				(0.024)			
*Lncentre×Invest*					-0.019		
					(0.027)		
*Lncentre×Insurance*						-0.026*	
						(0.014)	
*Lncentre×Money*							-0.016
							(0.019)
Controls	Yes	Yes	Yes	Yes	Yes	Yes	Yes
Year FE	Yes	Yes	Yes	Yes	Yes	Yes	Yes
N	25367	22176	22176	22176	18143	22176	13390

*Note*: ***, ** and * indicate significance at the 1%, 5% and 10% levels, respectively. *The clustered standard error at the city level is reported in parentheses*.

The results of column (2) show that the estimated coefficient of *Lncentre×Fintec* is negative, but does not pass the significance test, indicating that the regional development of financial technology does not have a significant impact on strengthening the “spatial spillover effects” of finance. Given that the digital technology empowering the financial industry is a systematic project encompassing multiple areas such as payments, investments, and more, and considering that the moderating effect of digitization may vary across different business areas, we decompose the China Digital Inclusive Finance Index and separately examine the impact of the application of financial technology in the fields of payment business (*Payment*), credit business (*Credit*), investment business (*Invest*), insurance business (*Insurance*), and money market fund business (*Money*) on the relationship between financial spatial structure and household financial vulnerability. The results are shown in columns (3) to (7) of **Table 12**. It can be observed that only the estimated coefficients of *Lncentre×Payment* and *Lncentre×Insurance* are significantly negative, while the coefficients of other interaction terms do not pass the significance tests.

The main reason is the differences of residents’ financial preferences. Payments, insurance, and credit are the most commonly financial activities that present in the production and daily life of families, and households have different demands for the digitization of these services. Payment services have lower risks, and the digitization of payment services saves space and time costs generated by traditional offline payments and settlement processes. It facilitates a large number of transactions that were previously suppressed due to remote and cumbersome issues [53], allowing households to monitor their financial situation timely. Residents can indiscriminately receive diffusive services from regional financial centers. Insurance services, with the dual functions of preservation and income, provide households with an effective risk management tool to cope with unexpected events, illnesses, property losses, and other risks. Whether online or offline, such products consistently occupy a prominent position in household financial activities. Digital technology has enhanced the sensitivity, effectiveness, and security of insurance product supply. The “spatial spillover effects” of finance provides households with more choices, better meeting their diverse needs. For credit services, although the development of financial technology has significantly reduced the non-performing loan ratio and management costs of banks [53], borrowers tend to obtain loans from the nearest banks geographically [57,58]. The increase in information search costs also restricts the speed and distance at which the service network of regional financial centers extends outward. It is observed that the application of digital technology in payment and insurance services can effectively strengthen the positive impact of financial “spatial spillover effects” on household financial vulnerability. However, the digitization of credit services does not show an obvious effect in leveraging the “spatial spillover effects” of finance.

## 7 Conclusion and insights

In recent years, the increase in household debt leverage and household financial vulnerability has posed practical challenges for preventing and controlling financial risks. Based on the panel data of Chinese Family Panel Studies (CFPS) and China’s cities from 2012–2020, we investigate the impact of financial spatial structure on household financial vulnerability from the perspectives of financial “local market effects” and “spatial spillover effects”. The findings reveal that: (1) both the “local market effects” and “spatial spillover effects” of finance effectively alleviate household financial vulnerability and the conclusions hold true even after addressing endogeneity issues, controlling for other influencing factors, and varying the measurement of variables. (2) The mechanisms by which financial spatial structure influences household financial vulnerability involve promoting household entrepreneurship, optimizing household asset allocation, and enhancing residents’ financial literacy. (3) Financial regulation and financial technology can strengthen the inhibitory effect of financial spatial structure on household financial vulnerability. By segmenting the application areas of financial technology, it is observed that the digitization of payment and insurance services can assist in alleviating household financial vulnerability through the “spatial spillover effects” of finance.

This paper provides a new research perspective for understanding household financial vulnerability, offering policy insights for alleviating household financial vulnerability and promoting high-quality financial development. Currently, a noticeable trend in China and various other emerging economies indicates a concentration of financial resources towards prominent cities and economic hubs. Simultaneously, there has been a rise in household leverage due to increasing housing loans and consumer credit. Given this context, the government must prioritize enhancing the financial system, promoting superior financial development, and attaining a harmonious equilibrium between sustainable growth and risk mitigation through strategic optimization of the financial framework. From the perspective of local governments, it is essential to further refine the financial spatial structure, optimize the supply of financial resources, and strengthen financial functions. Simultaneously, it is necessary to strengthen financial laws and regulations, enhance financial oversight, augment the transparency of the financial system, promptly identify and address potential risks, and ensure the stable and healthy development of financial markets. Efforts should be made to vigorously develop financial technology, utilizing technologies such as artificial intelligence and blockchain to enhance the automation, intelligence, convenience, and security of financial services. At the macroeconomic policy level, it is crucial to adopt a long-term perspective in managing the inflow of financial resources into economically developed regions. There should be efforts to expand the radiating and driving role of regional financial centers, promote complementary advantages, foster regional coordinated development, and achieve the transition from a financial power to a financial powerhouse.

The limitations inherent in this paper primarily manifest in two key areas. Firstly, the financial structure encompasses numerous facets, yet this study predominantly focused on elucidating the "local market effects" and "spatial spillover effects" of finance on household financial vulnerability, thereby neglecting other pertinent elements. Future scholarly inquiries have the potential to broaden the scope of the financial structure’s connotation and ascertain the ramifications of its alternate dimensions on the household sector. Secondly, while this paper principally analyzed the mechanisms by which the financial structure impacts household financial vulnerability from the perspective of household entrepreneurship, asset portfolio allocation, and residents’ financial literacy, future investigations could delve into alternative underlying mechanisms.

## Supporting information

S1 Data (DTA)
